# Supplementary material for: A Model of a Zebrafish Avatar for Co-Clinical Trials
Source: Cancers (Basel). 2020 Mar 13;12(3):677. doi: 10.3390/cancers12030677 (PMC7140063; doi:10.3390/cancers12030677)
Supplement: Supplementary file 1 [file cancers-12-00677-s001.zip › cancers-729951-final-supplementary/cancers-729951-layout-supplementary.docx]

Supplementary Materials

A Model of Zebrafish Avatar for Co-Clinical Trials

Alice Usai1, Gregorio Di Franco, Patrizia Colucci, Luca Emanuele Pollina, Enrico Vasile, Niccola Funel, Matteo Palmeri, Luciana Dente, Alfredo Falcone, Luca Morelli and Vittoria Raffa

**Table S1 Summary table o**f drugs used in the study.

| **Drug** | **Supplier** | **Class** |
| --- | --- | --- |
|  |  |  |
| 5-Fluorouracil | Teva | Antimetabolite |
| Cisplatin | Teva | Alkylating agent |
| Docetaxel | Accord HC | Mitotic inhibitors |
| Epirubicin | Teva | Antineoplastic agent |
| Gemcitabine | Hikma | Antimetabolite |
| Irinotecan | Hospira | Antineoplastic agent |
| Lederfolin | Pfizer | Antidote |
| Nab-paclitaxel | Celgene | Microtubule-targeting agent |
| Oxaliplatin | Teva | Alkylating agent |
|  |  |  |

**Table S2**. Chemotherapy protocol used in the study, clinical dose, corresponding plasma equivalent concentration (EPC) and concentrations tested in the zebrafish toxicity study (Figure 1). EPC was calculated as EPC = clinical dose x V/BSA, being V the reference value of the blood volume (5.4 L) and BSA the reference value of the body surface area (1.8 m^2^).

| **Chemotherapy Protocol** | **Drugs Combination** | **Clinical Dose  (mg/mq)** | **EPC  (mg/mL)** | **Concentration Used in Toxicity Assay (mg/mL)** | | | | |
| --- | --- | --- | --- | --- | --- | --- | --- | --- |
|  |  |  |  |  |  |  |  |  |
| **Gemcitabine** | Gemcitabine | 1000 | 0.34 | 0.05 | 0.10 | 0.25 | 0.50 |  |
|  |  |  |  |  |  |  |  |  |
| **GEMOX** | Gemcitabine | 1000 | 0.34 | 0.06 | 0.13 | 0.25 | 0.50 |  |
|  | Oxaliplatin | 100 | 0.03 | 0.01 | 0.01 | 0.03 | 0.05 |  |
|  |  |  |  |  |  |  |  |  |
| **GEM/*nab-*P** | Gemcitabine | 1000 | 0.34 | 0.06 | 0.08 | 0.13 | 0.25 |  |
|  | *nab*-Paclitaxel | 125 | 0.04 | 0.01 | 0.01 | 0.02 | 0.03 |  |
|  |  |  |  |  |  |  |  |  |
| **GEMCIS** | Gemcitabine | 1000 | 0.34 | 0.002 | 0.02 | 0.17 | 0.34 | 1.69 |
|  | Cisplatin | 25 | 0.01 | 0.00005 | 0.001 | 0.004 | 0.01 | 0.04 |
|  |  |  |  |  |  |  |  |  |
| **5-FU** | 5-Fluorouracil | 3200 | 1.08 | 1.08 | 2.70 | 5.39 | 10.80 |  |
|  |  |  |  |  |  |  |  |  |
| **FOLFOX** | 5-Fluorouracil | 2800 | 0.94 | 3.70 | 5.00 | 7.50 | 10.80 |  |
|  | Lederfolin | 200 | 0.07 | 0.26 | 0.36 | 0.54 | 0.77 |  |
|  | Oxaliplatin | 85 | 0.03 | 0.11 | 0.15 | 0.23 | 0.33 |  |
|  |  |  |  |  |  |  |  |  |
| **FOLFIRI** | 5-Fluorouracil | 2800 | 0.94 | 0.05 | 0.09 | 0.94 | 1.89 | 2.83 |
|  | Lederfolin | 200 | 0.07 | 0.004 | 0.01 | 0.07 | 0.14 | 0.20 |
|  | Irinotecan | 180 | 0.06 | 0.003 | 0.01 | 0.06 | 0.13 | 0.18 |
|  |  |  |  |  |  |  |  |  |
| **FOLFOXIRI** | 5-Fluorouracil | 3200 | 1.08 | 0.01 | 0.05 | 0.54 | 1.08 | 2.16 |
|  | Lederfolin | 200 | 0.07 | 0.001 | 0.003 | 0.03 | 0.070 | 0.14 |
|  | Oxaliplatin | 85 | 0.03 | 0.0003 | 0.001 | 0.01 | 0.030 | 0.06 |
|  | Irinotecan | 165 | 0.06 | 0.001 | 0.003 | 0.03 | 0.060 | 0.11 |
|  |  |  |  |  |  |  |  |  |
| **FLOT** | 5-Fluorouracil | 2600 | 0.88 | 0.10 | 0.20 | 0.30 | 0.40 | 0.50 |
|  | Lederfolin | 200 | 0.07 | 0.01 | 0.02 | 0.02 | 0.03 | 0.04 |
|  | Docetaxel | 50 | 0.02 | 0.002 | 0.01 | 0.01 | 0.01 | 0.02 |
|  | Oxaliplatin | 85 | 0.03 | 0.003 | 0.004 | 0.01 | 0.01 | 0.01 |
|  |  |  |  |  |  |  |  |  |
| **ECF** | 5-Fluorouracil | 2800 | 0.94 | 0.06 | 0.63 | 2.10 | 3.15 |  |
|  | Cisplatin | 60 | 0.02 | 0.001 | 0.01 | 0.05 | 0.07 |  |
|  | Epirubicin | 50 | 0.02 | 0.001 | 0.01 | 0.04 | 0.06 |  |

**
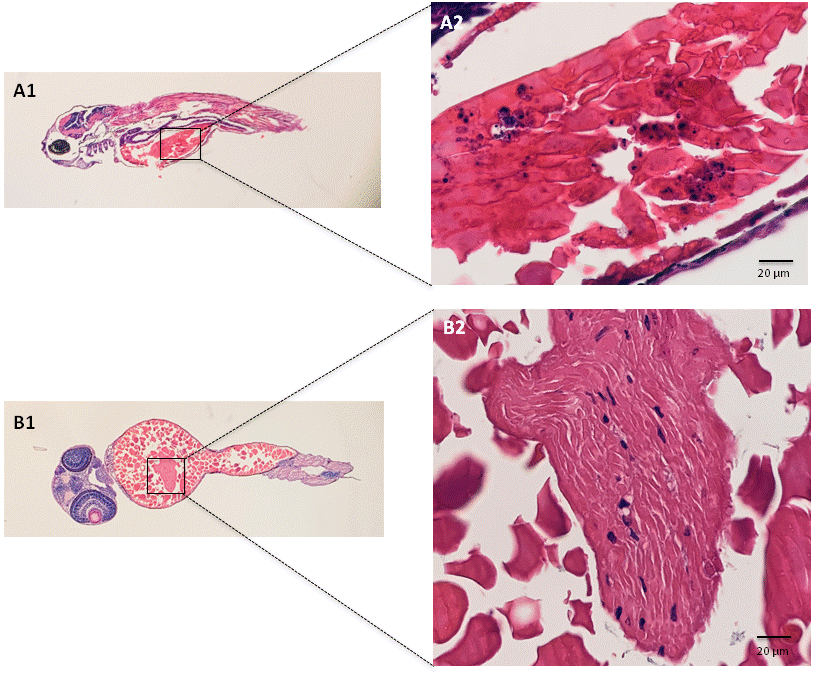
Figure S1.** H&E-stained sections of zebrafish embryo 2 days after receiving colon tumor tissue xenograft (**A1**–**A2**) and normal colon tissue xenograft (**B1**–***B2***) from the same patient.

**Figure S2.** Co-clinical trial. Establishment of a method for chemotherapy sensitivity testing. Human trial (black arrows). Patients are enrolled after informed consent in the study and underwent surgical resection. Excess tumor specimens not needed for clinical diagnosis are collected from the surgeon and screened by the histopathologist for selected adequate sampling of non-necrotic tumor. Patients receive a standard chemotherapy regimen and the oncologist is responsible to examine the follow-up of patients enrolled in the study. Animal trial (white arrows). Fresh patient tumor tissue is sectioned into ~3 mm^2^ pieces and fluorescent labeled. After processing, tumor pieces are implanted into the yolk sac of anaesthetized 2 dpf zebrafish embryos. Transplanted embryos are distributed in 24-multiwell plates. 2 hpi images are acquired of each xenotransplanted embryos in fluorescence and bright field. The following step is the exposition of zebrafish xenografts to different chemotherapy regimens to determine drug efficacy. The images are acquired and analyzed at 24 hpi and 48 hpi. The grey bar highlights the duration of the chemosensitivity assay.

**Video S1:** please view at the AVI fiel.

| 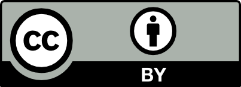 | © 2020 by the authors. Licensee MDPI, Basel, Switzerland. This article is an open access article distributed under the terms and conditions of the Creative Commons Attribution (CC BY) license (http://creativecommons.org/licenses/by/4.0/). |
| --- | --- |
